# Palytoxin Signal in LC-MS and UV: Preliminary Investigation on the Effect of Solvent and Temperature

**DOI:** 10.3390/toxins17060286

**Published:** 2025-06-06

**Authors:** Chiara Melchiorre, Michela Varra, Valeria Tegola, Valentina Miele, Carmela Dell’Aversano

**Affiliations:** 1 Department of Pharmacy, School of Medicine and Surgery, University of Naples Federico II, Via Domenico Montesano 49, 80131 Naples, Italy; valeria.tegola@unina.it (V.T.); valentina.miele@unina.it (V.M.); dellaver@unina.it (C.D.); 2 National Biodiversity Future Center, Piazza Marina 61, 90133 Palermo, Italy

**Keywords:** palytoxin, emerging toxins, LC-MRM-MS, mass spectrometry, UV-Vis spectrophotometry, solvent and temperature effect

## Abstract

Palytoxins (PLTXs) and ovatoxins (OVTXs) are a group of highly potent marine toxins that pose significant health risks through seafood contamination and environmental exposure. OVTX-producing algae have been linked to dermatitis and respiratory distress in Mediterranean beachgoers, while serious public health concerns are related to PLTX accumulation in seafood. In 2009, the European Food Safety Authority highlighted the need for analytical detection methods of the PLTX group of toxins and for the preparation of reference materials. This study investigates the stability of the palytoxin signal using liquid chromatography tandem mass spectrometry (LC-MRM-MS) and UV-Vis spectrophotometry under different experimental conditions: three concentrations (10, 1, and 0.5 µg/mL), three methanol–water mixtures (10%, 50%, and 90%), and two temperatures (6 °C and 25 °C). The results showed that the PLTX signal response is significantly influenced by the experimental conditions used. LC-MRM-MS analysis revealed the optimal response of PLTX in 50% and 90% MeOH at 25 °C, with minimal signal loss occurring over 16 h (9% and 6%). UV-Vis data indicated reduced absorbance in 10% MeOH, but a stable spectral intensity over 21 h in all the tested solvent mixtures. These results underscore the necessity of carefully controlled experimental conditions to ensure accurate and reproducible PLTX detection in environmental and food safety monitoring.

## 1. Introduction

**Figure 1:**
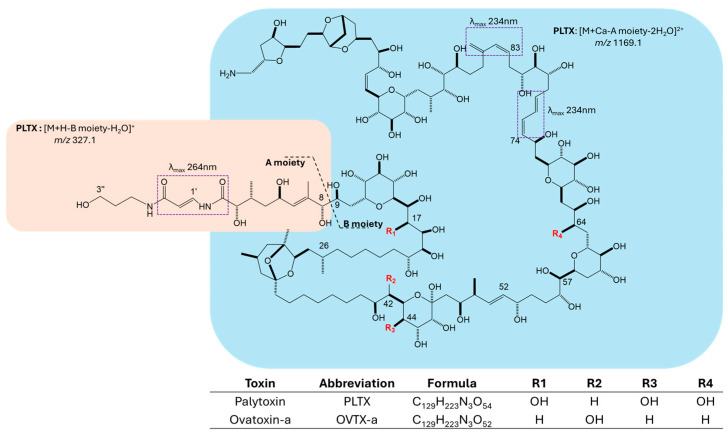
Structures of palytoxin [31] and OVTX-a [32]. Stereochemistry of OVTX-a is inverted at positions C-9, C-26, and C-57. Cleavage between carbons 8 and 9 originates A (orange box) and B (blue box) structural moieties which produce typical monocharged and double-charged ions in MS^2^ spectra: [M + H − B moiety − H_2_O]^+^ at *m*/*z* 327.1 (orange box) and [M + Ca − A moiety − 2H_2_O]^2+^ at *m*/*z* 1169.1 (blue box) [39]. Conjugated dienes responsible for the two absorption bands (λmax 264 and 234 nm) are highlighted in dashed boxes [41].

The palytoxin (PLTX) group of toxins includes highly potent marine biotoxins [1,2] from various species of zoanthids (*Palythoa* genus), dinoflagellates (*Ostreopsis* genus), and cyanobacteria (*Trichodesmium* genus) [3]. Ovatoxins (OVTXs) refer to a broad group of structural variants of PLTX produced by *Ostreopsis* cf. *ovata* and *O. fattorussoi*, with OVTX-a being the most prevalent congener in the Mediterranean strains [4,5] (Figure 1). During abundant *O.* cf. *ovata* blooms, the produced toxins present a potential risk for humans, who may become intoxicated through the inhalation of aerosolized toxins, direct skin/eye contact with contaminated waters, and/or the ingestion of contaminated seafood [6,7]. Since the late 1990s, hundreds of cases of respiratory distress and/or dermatitis have been recorded in people exposed to OVTX-producing algae (mainly *O.* cf. *ovata*) along the coasts of Italy, France, and Spain [6,8,9,10]. Outside of the Mediterranean area, *Ostreopsis* spp. were detected in coastal waters of Japan, New Zealand, Australia, and Brazil, among others [11,12,13,14,15,16,17,18,19,20]. The *Ostreopsis* phenomenon in Italy has been faced since its first dramatic onset during the Genoa outbreak in 2005 by clarifying many of the key issues for risk evaluation [21]. On that occasion, the presence of palytoxin congeners in the Mediterranean was ascertained for the first time, drawing up the complete toxin profile of *O.* cf. *ovata* [4,21]. A few years later, the accumulation of OVTXs in marine aerosols and seafood was confirmed [6,22], raising significant concerns for health protection. However, while the link between PLTXs in aerosols and respiratory distress in humans was demonstrated [23], most seafood poisonings that occurred in tropical regions are often linked to PLTXs based solely on clinical symptoms, with limited chemical evidence in support of the presence of toxins in food left-overs or biological fluids [16,24,25,26,27]. In the Mediterranean, although the PLTX group of toxins was found in various marine edible species, including mussels and sea urchins [8,22], no confirmed cases of human poisonings following ingestion of OVTX-contaminated seafood have been reported so far. Recently, in Brazil, a syndrome known as Haff Disease, characterized mainly by myalgia and rhabdomyolysis, was epidemiologically associated with the consumption of OVTX-contaminated fish [25].

Crucial aspects of the risk assessment of the PLTX group of toxins, such as hazard identification, exposure evaluation, and risk characterization, remain incomplete due to the lack of comprehensive toxicological data, limiting our understanding of PLTXs’ impact on human health [3,23]. This knowledge gap has contributed to the absence of regulatory measures by public health protection authorities. Currently, only five groups of marine biotoxins are regulated by the European Commission and the European Food Safety Authority (EFSA) [28,29] and thus are routinely monitored in seafood. Expanding surveillance lists to include emerging toxins such as PLTX and related compounds is essential. In 2009, the EFSA recommended a provisional maximum permitted level of 30 μg/kg for the total PLTX group of toxins in shellfish as a precautionary measure [29], underscoring the need for further research and regulatory action. This further highlights the importance of producing reference materials (RMs) for these compounds, which are essential for developing and validating analytical methods and biological assays and for conducting further toxicological studies.

The PLTX group of toxins exhibits minimal structural differences [4,14,30]. As an example, those related to the substitution pattern and stereochemistry between PLTX from *Palythoa* spp. [31] and OVTX-a from *O.* cf. *ovata* [32] are highlighted in Figure 1. This peculiarity makes PLTX discrimination in real samples particularly challenging, which is still of crucial importance. Indeed, individual congeners could present different levels of toxicity as a consequence of their different interaction with the main molecular target, namely Na^+^/K^+^ ATPase [33,34,35,36,37]. A recent study introduced a novel isolation procedure to obtain pure OVTX-a for analytical and toxicological applications [38]. However, to obtain a well-characterized and -quantified RM of each PLTX congener, namely solutions at a known concentration and composition, which is stable over time under defined conditions (temperature, etc.), the need for testing the PLTX response by different analytical techniques has emerged. Currently, the PLTX group of toxins is primarily detected and quantified using liquid chromatography coupled with tandem mass spectrometry (LC-MS-MS). They predominantly ionize in a positive mode, exhibiting peculiar ionization behavior and forming protonated molecular ions and adducts (e.g., sodium, potassium, or calcium) at different charge states [21,22,39,40]. UV-Vis spectrophotometry is also used for detecting the PLTX group of toxins based on their absorption bands centered at λ_max_ 234 and 264 nm, attributed to the presence of conjugated double bonds within their structure [41] (Figure 1). However, this technique is less sensitive than MS-based methods and requires higher toxin concentrations for effective detection [41,42,43].

This study aims to systematically investigate the instrumental response of PLTX under the solvent and temperature conditions commonly employed during the extraction and quantification of the PLTX group of toxins from biological matrices [16,43,44,45,46]. The outcomes shed light on the variability of the LC-MS response that, in turn, can affect PLTX quantitation both when this technique is used to analyze environmental and food samples [4,16,22] and when it is used to monitor the presence of PLTX congeners within isolation procedures [38,40].

**Figure 2:**
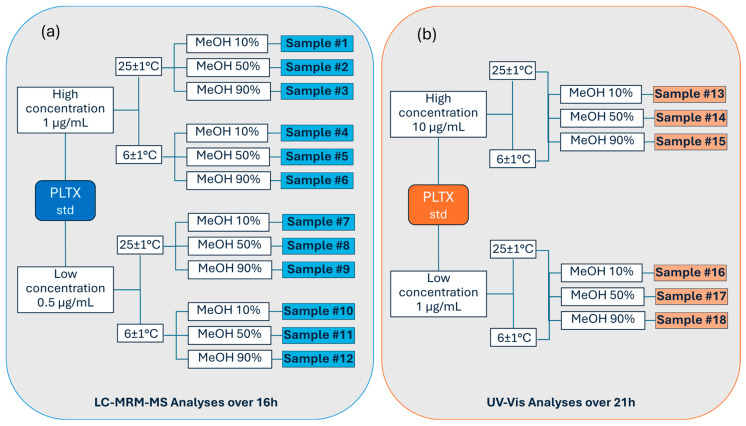
Experimental design used to assess the effect of temperature and solvent on PLTX instrumental response overtime by (**a**) LC-MRM-MS (0–16 h) and (**b**) UV-Vis (0–21 h).

The instrumental response of the PLTX standard was investigated using two different analytical techniques: LC-MS in a multiple-reaction monitoring mode (LC-MRM-MS) and UV-Vis spectrophotometry. For each technique, the PLTX standard was analyzed at relatively low and high levels of concentration, namely 0.5 (low) and 1 µg/mL (high) for LC-MRM-MS analyses, and 1 (low) and 10 µg/mL (high) for UV-Vis analyses. The experiments were performed at two operating temperature conditions (6 ± 1 and 25 ± 1 °C) in three aqueous methanol solvent mixtures, namely 10%, 50%, and 90% MeOH, in order to assess the temperature and solvent effect on the palytoxin signal response over time. The measurement values were recorded at multiple time points starting from the initial time (t_0_) and spanning up to 16 h (t_16_) and 21 h (t_21_) for LC-MRM-MS and UV-Vis, respectively. Figure 2 shows the experimental design, detailing the conditions used to analyze each sample by means of LC-MRM-MS (Figure 2a) and UV-Vis (Figure 2b).

The results offer important insights into the PLTX analytical behavior under these conditions, supporting the refinement of extraction and isolation methodologies. Moreover, the findings help to identify optimal handling conditions and contribute to enhancing the accuracy and reliability of quantification approaches for the PLTX group of toxins.

## 2. Results

### 2.1. Stability of LC-MRM-MS Signal Response

**Figure 3:**
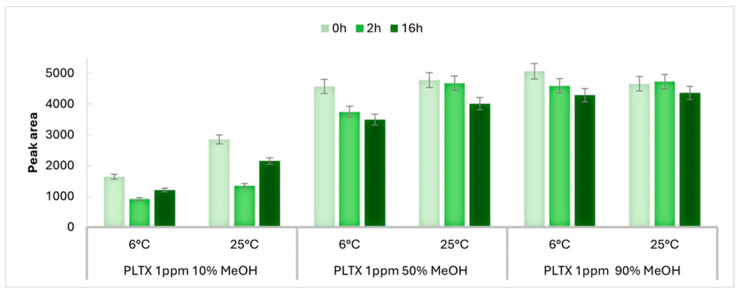
Comparison of peak area of transition *m*/*z* 906.9 → 327.1 of PLTX 1 µg/mL (1 ppm) acquired at time zero (0 h), after 2 h, and at 16 h in 10%, 50%, and 90% MeOH at 6 ± 1 and 25 ± 1 °C. Error bars represent the relative standard deviation of 5%, indicating the instrumental variability of measurements.

The influence of different methanol–water mixtures and temperatures on the PLTX signal response was assessed at two concentration levels (1 and 0.5 µg/mL) by monitoring the peak areas of the PLTX quantifier transition (906.9 → 327.1) over 16 h by means of the LC-MRM-MS method described in Section 5.3. Each of the samples #1–12 (Figure 2a) was analyzed soon after preparation to obtain the peak area at t_0_. Subsequently, each sample was analyzed at 2 h intervals over a total of 16 h. The instrumental responses obtained at each time point for the 1 µg/mL samples analyzed at 25 ± 1 and at 6 ± 1 °C are reported in Table S1 and Table S2, respectively. To identify trend variations in the instrumental response, the peak area at t_0_ for each sample was compared with that obtained at the first time point (t_2_), namely 2 h from sample preparation, and the last one (t_16_). The percentage variation in the peak area between time points t_0_ and t_16_ (Δt_0_–t_16_%) and, where necessary, between t_0_ and t_2_ (Δt_0_–t_2_%) were calculated. Figure 3 summarizes the results obtained for PLTX 1 µg/mL in 10%, 50%, and 90% MeOH at 25 ± 1 and 6 ± 1 °C.

At room temperature (25 ± 1 °C), samples in 50% and 90% MeOH exhibited the highest instrumental responses (peak area), nearly double that of the 10% MeOH sample, even at t_0_ (Figure 3 and Figure S1A–S3A, Table S1). The peak area of PLTX in 10% MeOH decreased significantly after 2 h (Δt_0_–t_2_% = 53%), reaching an equilibrium within 10 h, with an overall loss in signal of 25% being observed at t_10_ up to t_16_ (Table S1). Conversely, the peak area of PLTX in 50% and 90% MeOH showed excellent stability over time, exhibiting only 9% and 6% of peak area loss, respectively, over the 16 h period.

Even at 6 ± 1 °C, a typical temperature used in LC-MS auto-samplers when biological extracts are analyzed, the peak area at t_0_ of PLTX in 50% and 90% MeOH was significantly higher than that of PLTX in 10% MeOH (Figure 3 and Figure S1B–S3B, Table S2), which is consistent with the results observed at 25 ± 1 °C for the same level of concentration. In addition, for the PLTX sample in 10% MeOH, the peak area at t_0_ at 6 ± 1 °C was about 40% lower than that observed at 25 ± 1 °C (Figure 3 and Figure S1A,B, Table S1). At a low temperature, the PLTX signal response was negatively affected over time, especially in 10% and 50% MeOH solvent mixtures, as shown by the decreasing trend of the peak area over 16 h (Figure 3). Specifically, signal losses (Δt_0_–t_16_%) of 26%, 24%, and 15% were observed for PLTX in 10%, 50%, and 90% MeOH (Table S2), respectively.

**Figure 4:**
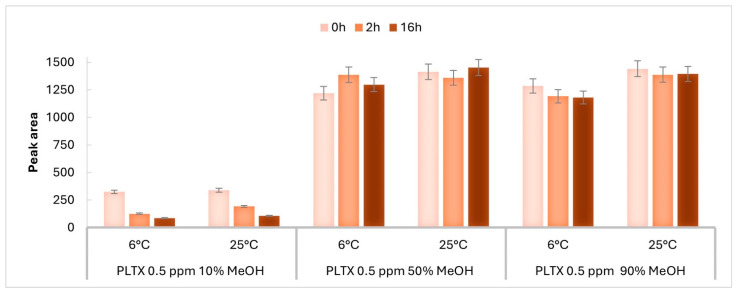
Comparison of peak area of transition *m*/*z* 906.9 → 327.1 of PLTX 0.5 µg/mL (0.5 ppm) acquired at time zero (0 h), after 2 h, and at 16 h, in 10%, 50% and 90% MeOH at 6 ± 1 and 25 ± 1 °C. Error bars represent the relative standard deviation of 5%, indicating the instrumental variability of measurements.

The above experiment was repeated using PLTX at a lower concentration level of 0.5 µg/mL (Figure 2a, Sample #7–12). The instrumental responses obtained at each time point at 25 ± 1 and at 6 ± 1 °C are reported in Table S3 and Table S4, respectively. The results (Figure 4) were consistent with those observed at the higher concentration level. For both operating temperatures, at t_0_, the peak area of PLTX in 50% and 90%MeOH was four times higher than that in 10%MeOH (Figure 4 and Figure S4–S6) and showed no significant change over the 16 h period. In contrast, the reduction in the peak area of PLTX in 10% MeOH over 16 h was even more pronounced compared to that observed at 1 µg/mL (Figure 3), with a peak area loss (Δt_0_−t_16_%) of 69% and 73% observed at 25 ± 1 and 6 ± 1 °C, respectively (Table S3 and Table S4).

### 2.2. Stability of PLTX UV-Vis Spectrophotometric Signal

Palytoxin contains a conjugated N-acylimine functionality at the A side of the molecule and two conjugated dienes at C74 and C83 (Figure 1). Accordingly, the UV-Vis spectrum of PLTX in water shows two absorption bands centered at λ 234 and 264 nm [41,42]. The molar extinction coefficients (ε) at the two λ_max_ for PLTX in water were reported as 47,000 (λ 233) and 28,000 (λ 263) [47].

To evaluate the effect of methanol–water mixtures (10%, 50% and 90% MeOH) and temperatures (25 ± 1 and 6 ± 1 °C) on PLTX UV-Vis absorption (Abs) properties, the spectra were monitored over time at two PLTX concentration levels (10 and 1 µg/mL) in the range 200–380 nm.

The Abs values at λ_max_ 234 and 264 nm were measured in Samples #13–18 (Figure 2b) soon after preparation (t_0_) and at 1 h intervals over a total of 21 h (t_21_) (Tables S5–S8).

**Figure 5:**
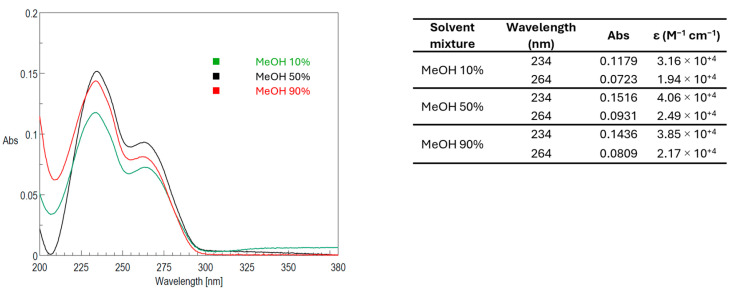
UV spectra of PLTX 10 µg/mL (3.73 × 10^−^^6^ M) acquired at time zero (t_0_) in 10% MeOH (green), 50% MeOH (black), and 90% MeOH (Red) at 25 ± 1 °C. Associated table reports molar extinction coefficients calculated at λ_max_ = 234 and 264 nm, respectively.

Figure 5 shows the UV spectra of the 10 µg/mL PLTX standard acquired at t_0_ in the three solvent mixtures and 25 ± 1 °C (Samples #13–15), with relevant molar extinction coefficients.

An appreciable difference was observed comparing PLTX Abs values in 10% MeOH (Figure 5, green line) with those in 50% and 90% MeOH (Figure 5, black and red lines, respectively). Indeed, the Abs values at λ_max_ 234 nm of PLTX in 50% and 90% MeOH were about 15% higher than that in 10% MeOH. This observation is consistent with the LC-MRM-MS data (Figure 3 and Figure 4). An analysis of the spectra acquired over 21 h revealed that Abs values remained largely unchanged (Δt_0_−t_21_ ≤ 5%) across all tested solvent mixtures (Table S5).

**Figure 6:**
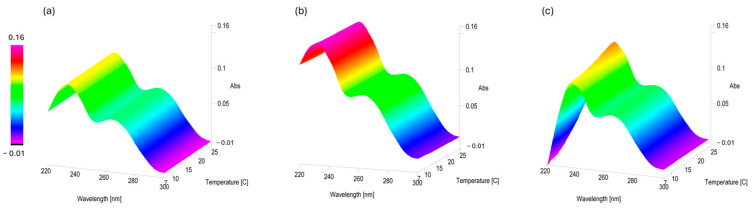
UV-Vis thermal scan interval data analysis from 25 to 7 °C, 0.5 °C/min, of PLTX 10 µg/mL samples in 10% (**a**), 50% (**b**), and 90% MeOH (**c**).

The same set of PLTX 10 µg/mL samples were cooled down by gradually reducing the operating temperature from 25 to 7 °C at a rate of 0.5 °C/min, with spectra recorded every minute during the cooling phase (Figure 6). Subsequently, the spectra were recorded hourly over a 21 h period while maintaining a constant temperature at 6 ± 1 °C.

**Figure 7:**
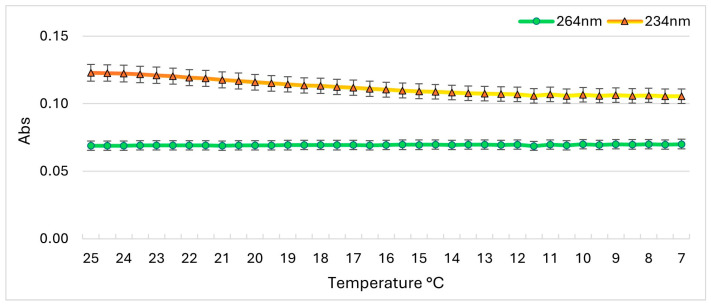
Abs at 234 and 264 nm recorded for PLTX 10 µg/mL in 90% MeOH during the cooling process from 25 to 7 °C, 0.5 °C/min. Error bars represent the standard deviation of 5%, indicating the instrumental variability of measurements.

PLTX 10 µg/mL samples in 10% and 50% MeOH (Figure 6a,b) did not show any significant variation in the Abs values at λ_max_ 234 and 264 nm. In contrast, the PLTX 10 µg/mL sample in 90% MeOH showed a visible decrease in the Abs value at λ_max_ 234 nm, as evidenced in Figure 6c by the peak color changes from orange to yellow. Indeed, as can be seen by plotting the Abs values during the cooling process versus temperature (Figure 7), a gradual decay of Abs at λ_max_ 234 nm occurred as the temperature decreased from 25 to 12 °C, stabilizing between 12 and 7 °C. In contrast, the Abs value at λ_max_ 264 nm remained constant throughout the cooling process.

Once the temperature reached 6 ± 1 °C, the Abs at 234 and 264 nm were recorded every hour for 21 h (Table S6). In all samples, Abs values remained largely unchanged (Δt_0_−t_21_ ≤ 5%).

**Figure 8:**
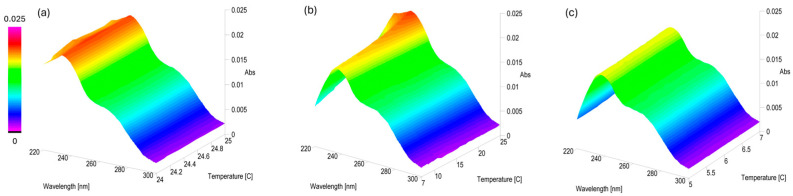
UV-Vis thermal scan interval data analysis for PLTX 1 µg/mL in 50% MeOH (**a**) from 24 to 25 °C, 0.1 °C/hour; (**b**) from 25 to 7 °C, 0.5 °C/min; and (**c**) from 7 to 5 °C, 0.1 °C/hour.

The above experiment was repeated using PLTXs at a lower concentration level of 1 µg/mL. In 10% and 90% MeOH, the characteristic UV-Vis profile of palytoxins was no longer observed under any of the tested temperature conditions. The band centered at 234 nm was fully overlapped with that of the solvent, while the band centered at 264 nm was barely visible. This made the data interpretation unreliable for these samples (Samples #16 and 18, Figure 2b). In contrast, PLTX 1 µg/mL in 50% MeOH at 25 ± 1 °C exhibited largely unchanged Abs values in all spectra acquired over 21 h (Figure 8a, Table S7).

Subsequently, the operating temperature gradually lowered by 0.5 °C/min from 25 to 7 °C, with spectra acquired every minute during the cooling phase. An appreciable decrease (>15%) in the Abs value at λ_max_ 234 nm was observed as the temperature dropped down from 25 to 12 °C (Figure 8b), stabilizing between 12 and 7 °C. The analysis of the spectra at 6 ± 1 °C over 21 h showed that, despite the initial decrease, the absorbance values at both λ_max_ remained nearly constant once the sample reached 6 ± 1 °C (Δt_0_−t_21_ ≤ 5%) (Figure 8c, Table S8).

## 3. Discussion

The results from both UV-Vis and LC-MRM-MS analyses suggest that the water percentage in the solvent mixture and operating temperature are critical factors influencing the analytical response of palytoxin. Using LC-MRM-MS, the mostly aqueous solvent mixture (10% MeOH), leads to a much lower PLTX peak area than those observed in the other two solvent mixtures at both operating temperatures and to more pronounced changes in the instrumental response over 16 h. These effects increase as the PLTX concentration level decreases (Figure 3 and Figure 4).

The highest LC-MRM-MS signal responses were achieved when PLTX was dissolved in mid-to-high proportions of organic solvent (50% and 90% MeOH) and analyzed at 25 °C (Figure 3). These conditions likely promote more efficient PLTX ionization by reducing solvation-related effects—such as conformational changes or molecular aggregation [40,48,49]—that could affect the abundance of the multicharged adducts, including the targeted [M + H + Ca]^3+^ ion. Indeed, PLTX exhibits a complex full-scan mass spectrum characterized by the presence of multiple-charged metalated ions (2+, 3+, and 4+), protonated ions (1+, 2+, and 3+), in-source fragments (1+ and 2+)—mainly due to cleavage at C8-C9 (Figure 1)—as well as dimers and trimers (3+ and 5+), most of which undergo multiple instances of water loss (Figure S7C) [21,39,40]. This MS behavior reflects the large molecular size and structural complexity of PLTX and points out the critical role that solvents may play in determining the relative abundance of the ion species formed in an aqueous solution and/or in the ionization source [40,49].

Moreover, for PLTX samples in 50% and 90% MeOH, the obtained results revealed a moderate but still appreciable influence of lowering the operating temperature at 6 °C on the PLTX peak area over time (Figure 3 and Figure 4). This suggests that, although temperature is not the primary driver for variation in the PLTX signal, it should be taken into account for PLTX detection and quantification under the tested solvent conditions.

Optimizing the water percentage in solvent mixtures as well as the operating temperatures is thus important, especially in applications where the quantification accuracy and analytical reproducibility are essential. The influence of the solvent composition is especially relevant during complex laboratory workflows such as extraction, isolation, and purification, where samples are often exposed to varying solvent mixtures and temperatures over extended periods [38,45,50]. In these scenarios, temperature fluctuations can intensify solvent effects, especially at low MeOH concentrations.

The UV-Vis spectroscopy data support the LC-MS findings. At room temperature, PLTX dissolved in 10% MeOH showed a weaker absorbance profile compared to the 50% and 90% methanol solutions (Figure 5), suggesting increased molecular aggregation in predominantly aqueous environments [40,48,49]. Despite these differences in intensity, the UV-Vis absorbance bands remained relatively stable over time in all the tested solvent mixtures, indicating no significant degradation of the toxin during the observational period (Tables S5 and S6).

However, temperature also had a pronounced effect on UV-Vis profiles (Figure 6 and Figure 7), particularly in samples solubilized in higher MeOH concentrations (90%MeOH). The effect was more pronounced in lowering the PLTX concentration from 10 to 1 µg/mL. Indeed, the UV-Vis profile of PLTX 1 µg/mL was detectable only in the 50%MeOH sample (Figure 8). These observations likely reflect solvent- and temperature-induced conformational rearrangements [51,52].

When UV-Vis is employed as a detector in LC systems using an organic-water-gradient elution, the observed solvent-induced effects could affect the analytical performance. The characteristic UV band of PLTX centered at 234 nm (associated with the highest molar extinction coefficient) may be partially masked by the mobile phase absorbance, particularly during transitions from aqueous to organic-rich eluents. This could result in signal suppression or baseline instability, complicating the detection and quantification.

Our findings underscore that a predominantly aqueous solvent is not recommended for preparing the PLTX standard. Solvation phenomena through hydrogen bonding, dipole–dipole interactions, or hydrophobic interactions may promote conformational changes and/or molecular aggregation. These effects are consistent with previous reports on similar large, hydrophilic marine toxins that exhibit solvation-dependent conformational variability, impacting their behavior in mass spectrometry and chromatography [40,48,49]. Nevertheless, in biological assays or in vivo studies where the use of predominantly aqueous solutions is often unavoidable, our findings suggest that the PLTX standard should be equilibrated at room temperature (25 °C) for at least four hours before analysis. This equilibration time may stabilize the molecular conformation resulting in more consistent and reliable results.

## 4. Conclusions

This preliminary study demonstrates that the commonly used aqueous methanol solvent mixtures and operating temperatures influence both the LC-MS and UV-Vis signal intensity in a manner that is dependent on the concentration of PLTX, with more pronounced effects observed at relatively low concentrations and temperatures. Thorough control over the solvent and temperature during all phases of PLTX handling and analysis is thus needed.

Future in-depth research should also expand the present findings by investigating PLTX behavior under complex matrix conditions and over extended timeframes. Alternative solvents, stabilizers, or storage technologies may further enhance the PLTX stability and analytical performance and thus should be explored. The investigation of PLTX long-term stability is critical not only for ensuring proper sample preservation, but also to facilitate the production of reference materials for PLTX-like compounds, which are currently lacking on the market but are still essential for method validation, regulatory monitoring, and in vivo toxicological studies.

Ultimately, this study represents a crucial first step towards a more comprehensive understanding of PLTX and related marine toxins. Looking forward, the improvement and reliability of PLTX detection and the reproducibility of the analytical method will directly contribute to public health protection, enabling a quicker response to toxic outbreaks and improved risk assessments.

## 5. Materials and Methods

### 5.1. Materials

PLTX standard 100 µg (Lot. N° WTR7399) was purchased from FUJIFILM Wako Chemicals Europe GmbH (Neuss, Germany) and stored at −20 °C until sample preparation. MilliQ water (W) was obtained by double filtration by Sinergy UV LC-Pak Polisher (Lot. N° F3NB63201) purchased from Merck Millipore (Darmstadt, Germany), Methanol (MeOH) and Acetonitrile (ACN) were purchased from VWR International Srl (Milan, Italy), and Acetic Acid (AA) was provided by Merck Sigma-Aldrich (Darmstadt, Germany). Quartz cuvettes with optical path 1 cm and max capacity 700 µL were purchased from Hellma Italia S.r.l. (Milan, Italy).

### 5.2. Sample Preparation for LC-MRM-MS Analyses

PLTX standard 100 µg was added to 2 mL 50% aqueous MeOH, mixed, and left at room temperature for 5 h (PLTX stock solution, 50 µg/mL). The stock solution was diluted 1:5 with 50% MeOH (PLTX, 10 µg/mL) and, immediately before starting the analysis, further diluted with different volumes of MeOH and/or water to obtain PLTX samples 1 and 0.5 µg/mL in 10%, 50%, and 90% aqueous MeOH. Samples #1–6 (Figure 2a) were prepared by 1:10 dilution of PLTX 10 µg/mL (Table S9), while Samples #7–12 (Figure 2a) were prepared by a two-step dilution, a 1:10 dilution of PLTX 10 µg/mL (Table S9) followed by a 1:2 dilution of PLTX 1 µg/mL (Table S10).

### 5.3. LC-MRM-MS Method

LC-MRM-MS experiments were carried out on a Triple Quadrupole MS Agilent G6470 (SN: SG2050G208, Agilent, Santa Clara, CA 95051 USA ) equipped with an ESI ION source and coupled with an ultra-high-performance liquid chromatography (UHPLC) Agilent 1290 Infinity II (SN: DEBAZ05437, Agilent, Santa Clara, CA 95051 USA). Target acquisition was carried out with Agilent MassHunter Qualitative and Quantitative Analysis software V10.0 (2006–2018). Optimal ion source parameters consisted of Gas Temperature (°C) 350, Gas Flow (L/min) 12, Nebulizer pressure (psi) 30, Sheath Gas Temperature (°C) 350, Sheath Gas flow (L/min) 11, Capillary current (nA) 4500 and Nozzle Voltage 1000 (V). Experiments were carried out in MRM mode (positive ions), scanning two transitions precursor ion → product ion. Collision-Induced Dissociation (CID) was set at 30 eV. The [M + H + Ca]^3+^ ion of PLTX at *m*/*z* 906.9 was selected as precursor ion of the transitions to the following product ions [M + H − B moiety − H_2_O]^+^ at *m*/*z* 327.1 (Transition #1: 906.9 → 327.1) and [M + Ca − A moiety − 2H_2_O]^2+^ at *m*/*z* 1169.1 (Transition #2: 906.9 → 1169.1), with the former used as Quantifier transition. These transitions were selected based on previous data [39], after checking the presence of the precursor ion in full-scan MS spectrum (Figure S7). Optimization of source and acquisition parameters was conducted directly in MRM mode using a PLTX standard.

**Figure 9:**
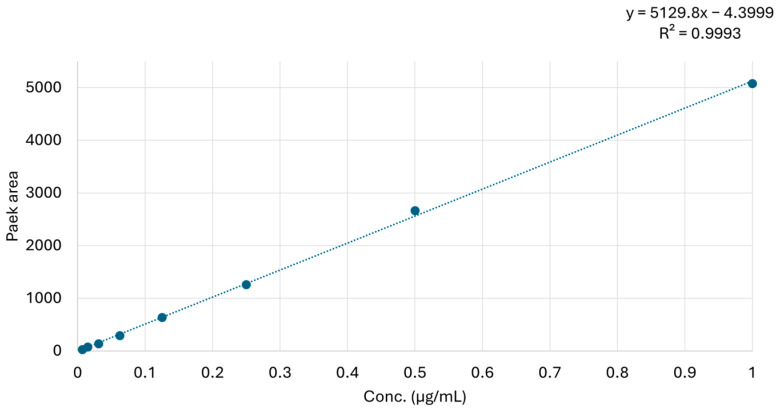
Eight-level calibration curve of PLTX standard in 50% MeOH (1, 0.5, 0.25, 0.125, 0.0625, 0.0313, 0.0156, and 0.0078 μg/mL) with graph equation and R^2^.

Chromatographic separation was achieved on a C18 Kinetex 2.6 µm 100 Å 100 × 2.1 mm column (Phenomenex, Torrance CA, USA) using a binary mobile phase consisting of water 100% (eluent A) and aqueous acetonitrile 95% (eluent B), both containing 30 mM acetic acid. The elution gradient rose from 20% to 100% of B in 10 min. After 5 min of hold time at 100% B, 20% B was reached within 1 min, followed by 10 min re-equilibration at 20% B. The total chromatographic run time was 26 min. The column temperature was maintained at 25 ± 1 °C, injection volume was 5 µL, and flow rate was 0.2 mL/min. The LC autosampler (Agilent, Santa Clara, CA 95051 USA) was kept at the operating temperatures of 6 ± 1 or 25 ± 1 °C during the experiments. The two concentration levels of PLTX standard (1 and 0.5 µg/mL) were selected based on linearity of the response (peak area) observed for Transition #1 within the range 0.007 to 1 µg/mL of PLTX in 50% MeOH (Figure 9).

### 5.4. Samples Preparation for UV-Vis Analyses

For UV-Vis spectrophotometric study, 6 PLTX samples were prepared by diluting PLTX stock solution (50 µg/mL) immediately before analysis with different volumes of MeOH and/or water to obtain PLTX 10 and 1 µg/mL in 10%, 50%, and 90% aqueous MeOH. Samples #13–15 (Figure 2b) were prepared by 1:5 dilution of PLTX 50 µg/mL (Table S11), while Samples #16–18 (Figure 2b) were prepared by a two-step dilution: a 1:5 dilution of PLTX 50 µg/mL followed by a 1:10 dilution of PLTX 10 µg/mL (Table S12).

### 5.5. UV-Vis Spectrophotometry Method

UV-Vis experiments were carried out by using a Jasco V750 Spectrophotometer equipped with temperature-controlled cell (Jasco Europe S.R.L, Cremella, Italy). All spectra were acquired in the wavelength range of 380–200 nm, band width of 2 nm, data interval of 0.5 nm, UV-Vis response of 0.06 sec, and scan speed of 400 nm/min. For experiments at 25 ± 1 and at 6 ± 1 °C, the temperature was kept constant by a temperature control system and by using N2 steam to avoid condensation interference. Spectra were acquired for each sample every hour over a total of 21 h. For each experiment, the UV-Vis spectrum of the empty cell (baseline), of the aqueous methanol solution (blank), and of the sample were acquired in sequence (Table S13). Before proceeding with the experiment at 6 ± 1 °C, the temperature was decreased gradually to 7 °C. The instrument was set in ScanT modality, acquiring spectra every 0.5 °C/min with a temperature ramp ranging from 25 °C to 7 °C.

## Data Availability

The original contributions presented in this study are included in the article/Supplementary Materials. Further inquiries can be directed to the corresponding author(s).

## Supplementary Materials

The following supporting information can be downloaded at: https://www.mdpi.com/article/10.3390/toxins17060286/s1, Table S1: LC-MRM MS peak area of PLTX 1 µg/mL obtained at each time point 0 h–16 h at 25 ± 1 °C. Table S2: LC-MRM MS peak area of PLTX 1 µg/mL obtained at each time point 0 h–16 h at 6 ± 1 °C. NA = not acquired due to an injection issue. Table S3: LC-MRM MS peak area of PLTX 0.5 µg/mL obtained at each time point 0 h–16 h at 25 ± 1 °C. Table S4: LC-MRM MS peak area of PLTX 0.5 µg/mL obtained at each time point 0 h–16 h at 6 ± 1 °C. NA = not acquired due to an injection issue. Table S5: UV-Vis absorbances at 234 nm and 264 nm of PLTX 10 µg/mL obtained at time zero (t_0_) and after 21 h (t_21_) with relative variation (Δ Abs%) at 25 ± 1 °C. Table S6: UV-Vis absorbances at 234 nm and 264 nm of PLTX 10 µg/mL obtained at time zero (t_0_) and after 21 h (t_21_) with relative variation (Δ Abs%) at 6 ± 1 °C. Table S7: UV-Vis absorbances at 234 nm and 264 nm of PLTX 1 µg/mL obtained at time zero (t_0_) and after 21 h (t_21_) with relative variation (Δ Abs%) at 25 ± 1 °C. Table S8: UV-Vis absorption values measured for PLTX 1 µg/mL at 6 ± 1 °C, λ_max_ 234 nm and 264 nm, time zero (t_0_) and after 21 h (t_21_) with relative variation (Δ Abs%). Table S9: Procedure used for preparation of Sample #1–6. Table S10: Procedure used for preparation of Sample #7–12. Table S11: Procedure used for preparation of Sample #13–15. Table S12: Procedure used for preparation of Sample #16–18. Table S13: Example of the procedure used to acquire UV-Vis spectra for Sample #13 and #14, namely PLTX 10 µg/mL in 10% MeOH at 25 °C and PLTX 10 µg/mL in 50% MeOH at 25 °C. Figure S1: Total Ion Chromatogram (TIC) and MRM quantifier transition (906.9 ± 327.1) for PLTX 1 µg/mL in 10%MeOH at t_0_ at 25 ± 1 °C (A) and 6 ± 1 °C (B). Figure S2: Total Ion Chromatogram (TIC) and MRM quantifier transition (906.9 ± 327.1) for PLTX 1 µg/mL in 50%MeOH at t_0_ 25 ± 1 °C (A) and 6 ± 1 °C (B). Figure S3: Total Ion Chromatogram (TIC) and MRM quantifier transition (906.9 ± 327.1) for PLTX 1 µg/mL in 90%MeOH at t_0_ 25 ± 1 °C (A) and 6 ± 1 °C (B). Figure S4: Total Ion Chromatogram (TIC) and MRM quantifier transition (906.9 ± 327.1) for PLTX 0.5 µg/mL in 10%MeOH at t_0_ 25 ± 1 °C (A) and 6 ± 1 °C (B). Figure S5: Total Ion Chromatogram (TIC) and MRM quantifier transition (906.9 ± 327.1) for PLTX 0.5 µg/mL in 50%MeOH at t_0_ 25 ± 1 °C (A) and 6 ± 1 °C (B). Figure S6: Total Ion Chromatogram (TIC) and MRM quantifier transition (906.9 ± 327.1) for PLTX 0.5 µg/mL in 90%MeOH at t_0_ 25 ± 1 °C (A) and 6 ± 1 °C (B). Figure S7: LC-MS of PLTX 1 µg/mL in 50% MeOH acquired in full-scan MS positive ion mode in the mass range *m*/*z* 500–1500, scan time 500 ms, fragmentor 135 V, accelerator voltage 5 V. (A) Total ion chromatogram (TIC), (B) extracted ion chromatogram (EIC) of [M + H + Ca]^3+^ ion of palytoxin at *m*/*z* 906.9, and (C) full-scan MS spectrum associated with the peak eluting at 3.5 min with ion assignment of the main ions.

## Abbreviations

The following abbreviations are used in this manuscript:

AA	Acetic acid
Abs	Absorption
ACN	Acetonitrile
CID	Collision-Induced Dissociation
EFSA	European Food Safety Authority
LC	Liquid chromatography
LC-MS-MS	Liquid chromatography coupled with tandem mass spectrometry
MeOH	Methanol
MRM	Multiple-reaction monitoring mode
MS	Mass spectrometry
OVTX	Ovatoxin
PLTX	Palytoxin
RM	Reference Material
UHPLC	Ultra-high-performance liquid chromatography
UV-Vis	Ultraviolet-visible spectrophotometry
W	MilliQ water
