# Supplementary material for: Palytoxin Signal in LC-MS and UV: Preliminary Investigation on the Effect of Solvent and Temperature
Source: Toxins (Basel). 2025 Jun 6;17(6):286. doi: 10.3390/toxins17060286 (PMC12197661; doi:10.3390/toxins17060286)
Supplement: Supplementary file 1 [file toxins-17-00286-s001.zip › toxins-3658599-supplementary.pdf]

Supplementary material: Palytoxin Signal in LC-MS and UV: Preliminary Investigation on the Effect of Solvent and Temperature

Chiara Melchiorre, Michela Varra, Valeria Tegola, Valentina Miele and Carmela Dell'Aversano

Table S1 *LC-MRM MS peak area of PLTX 1 μg/mL obtained at each time point 0h-16h at 25±1°C.*

**Time point**	**Time (h)**	**Peak area PLTX 1 μg/mL at 25±1°C**		
		**10%MeOH**	**50%MeOH**	**90%MeOH**
t_0	0	2848,60	4770,95	4658,07
t_2	2	1347,22	4676,19	4725,96
t_4	4	1295,24	4084,70	4431,24
t_6	6	1407,64	4246,24	4494,95
t_8	8	1812,64	4269,84	4645,68
t_{10}	10	2120,22	4238,36	4041,50
t_{12}	12	2149,24	4198,68	4592,14
t_{14}	14	2087,12	4234,59	4341,33
t_{16}	16	2147,90	4344,53	4358,56
$\Delta_{\text{Area}}\,\% = \frac{(t0-t16)}{t0} \times 100$		$\Delta_{\text{Area}} = 25\%$	$\Delta_{\text{Area}} = 9\%$	$\Delta_{\text{Area}} = 6\%$

Table S2 *LC-MRM MS peak area of PLTX 1 μg/mL obtained at each time point 0h-16h at 6±1°C. NA=not acquired due to an injection issue.*

**Time point**	**Time (h)**	**Peak area PLTX 1 μg/mL at 6±1°C**		
		**10%MeOH**	**50%MeOH**	**90%MeOH**
t_0	0	1634,76	4567,04	5057,37
t_2	2	910,34	3745,61	4586,83
t_4	4	1144,40	NA	4586,83
t_6	6	1178,00	3865,59	4856,78
t_8	8	1231,90	4281,72	4691,41
t_{10}	10	1267,41	3720,38	4365,06
t_{12}	12	1251,51	3541,64	4396,43
t_{14}	14	1330,42	3662,02	4312,88
t_{16}	16	1202,12	3492,32	4287,63
$\Delta_{\text{Area}}\,\% = \frac{(t0-t16)}{t0} \times 100$		$\Delta_{\text{Area}} = 26\%$	$\Delta_{\text{Area}} = 24\%$	$\Delta_{\text{Area}} = 15\%$

Table S3 *LC-MRM MS peak area of PLTX 0.5 µg/mL obtained at each time point 0h-16h at 25±1°C.*

Time point	Time (h)	Peak area PLTX 0.5 µg/mL at 25±1°C		
		10%MeOH	50%MeOH	90%MeOH
t_0	0	338,06	1412,69	1441,70
t_2	2	191,80	1359,57	1387,66
t_4	4	117,82	1442,33	1443,14
t_6	6	119,45	1314,59	1241,21
t_8	8	125,64	1521,90	1481,02
t_{10}	10	133,25	1487,38	1414,82
t_{12}	12	127,83	1533,85	1571,74
t_{14}	14	98,78	1539,00	1590,65
t_{16}	16	106,07	1451,92	1394,16
$\Delta_{\text{Area}}\,\% = \frac{(t0-t16)}{t0} \times 100$		Δ_{Area} = 69%	Δ_{Area} = -3%	Δ_{Area} = 3%

Table S4 *LC-MRM MS peak area of PLTX 0.5 µg/mL obtained at each time point 0h-16h at 6±1°C. NA=not acquired due to an injection issue.*

Time point	Time (h)	Peak area PLTX 0.5 µg/mL at 6±1°C		
		10%MeOH	50%MeOH	90%MeOH
t_0	0	323,17	1218,71	1285,94
t_2	2	125,47	1387,25	1192,25
t_4	4	95,56	1251,94	1140,5
t_6	6	117,37	NA	1021,67
t_8	8	74,83	1251,06	1092,61
t_{10}	10	135,67	1263,04	1072,23
t_{12}	12	79,77	1226,69	1113,23
t_{14}	14	75,44	1324,47	1060,78
t_{16}	16	86,37	1297,21	1180,61
$\Delta_{\text{Area}}\,\% = \frac{(t0-t16)}{t0} \times 100$		Δ_{Area} = 73%	Δ_{Area} = -6%	Δ_{Area} = 8%

Table S5 *UV-Vis absorbances at 234nm and 264nm of PLTX 10 µg/mL obtained at time zero (t_0) and after 21h (t_{21}) with relative variation (Δ Abs %) at 25±1°C.*

**Time point**	**Time (min)**	**UV-Abs of PLTX 10 µg/mL at 25±1°C**					
		**10%MeOH**		**50%MeOH**		**90%MeOH**	
		λ_{264nm}	λ_{234nm}	λ_{264nm}	λ_{234nm}	λ_{264nm}	λ_{234nm}
t_0	0	0,0662	0,1115	0,0931	0,1516	0,0704	0,1263
t_1	60	0,0662	0,1116	0,0886	0,1557	0,0704	0,1263
t_2	120	0,0663	0,1117	0,0885	0,1556	0,0701	0,1260
t_3	180	0,0664	0,1116	0,0883	0,1555	0,0698	0,1248
t_4	240	0,0658	0,1115	0,0882	0,1556	0,0697	0,1245
t_5	300	0,0658	0,1114	0,0885	0,1554	0,0694	0,1242
t_6	360	0,0658	0,1111	0,0884	0,1553	0,0694	0,1242
t_7	420	0,0657	0,1110	0,0883	0,1553	0,0693	0,1239
t_8	480	0,0656	0,1109	0,0884	0,1553	0,0691	0,1237
t_9	540	0,0653	0,1108	0,0883	0,1553	0,0692	0,1236
t_{10}	600	0,0632	0,1062	0,0886	0,1554	0,0690	0,1234
t_{11}	660	0,0638	0,1070	0,0887	0,1554	0,0688	0,1234
t_{12}	720	0,0640	0,1077	0,0886	0,1552	0,0688	0,1231
t_{13}	780	0,0646	0,1086	0,0887	0,1553	0,0686	0,1230
t_{14}	840	0,0651	0,1094	0,0886	0,1553	0,0686	0,1230
t_{15}	900	0,0662	0,1116	0,0886	0,1552	0,0687	0,1228
t_{16}	960	0,0632	0,1063	0,0888	0,1553	0,0686	0,1227
t_{17}	1020	0,0632	0,1064	0,0888	0,1552	0,0686	0,1226
t_{18}	1080	0,0633	0,1066	0,0887	0,1552	0,0687	0,1226
t_{19}	1140	0,0633	0,1066	0,0888	0,1553	0,0684	0,1226
t_{20}	1200	0,0633	0,1063	0,0888	0,1556	0,0684	0,1224
t_{21}	1260	0,0632	0,1065	0,0887	0,1555	0,0682	0,1222
$\Delta_{Abs}\% = \frac{(t0-t21)}{t0} \times 100$		Δ_{Abs}= 4%	Δ_{Abs}= 5%	Δ_{Abs}= 5%	Δ_{Abs}= 2%	Δ_{Abs}= 3%	Δ_{Abs}= 3%

Table S6 *UV-Vis absorbances at 234nm and 264nm of PLTX 10 µg/mL obtained at time zero (t_0) and after 21h (t_{21}) with relative variation (Δ Abs %) at 6±1°C.*

**Time point**	**Time (min)**	**UV-Abs of PLTX 10 µg/mL at 6±1°C**					
		**10%MeOH**		**50%MeOH**		**90%MeOH**	
		λ_{264nm}	λ_{234nm}	λ_{264nm}	λ_{234nm}	λ_{264nm}	λ_{234nm}
t_0	0	0,0643	0,1065	0,0738	0,1257	0,0699	0,1068
t_1	60	0,0642	0,1062	0,0734	0,1241	0,0699	0,1068
t_2	120	0,0642	0,1060	0,0730	0,1236	0,0703	0,1102
t_3	180	0,0641	0,1059	0,0727	0,1230	0,0699	0,1096
t_4	240	0,0641	0,1059	0,0724	0,1223	0,0699	0,1097
t_5	300	0,0641	0,1059	0,0722	0,1220	0,0695	0,1093
t_6	360	0,0639	0,1058	0,0717	0,1219	0,0693	0,1087
t_7	420	0,0641	0,1058	0,0720	0,1215	0,0690	0,1082
t_8	480	0,0641	0,1057	0,0717	0,1211	0,0687	0,1079
t_9	540	0,0640	0,1056	0,0713	0,1207	0,0687	0,1074
t_{10}	600	0,0638	0,1054	0,0711	0,1203	0,0685	0,1076
t_{11}	660	0,0639	0,1056	0,0710	0,1200	0,0683	0,1070
t_{12}	720	0,0640	0,1055	0,0709	0,1198	0,0683	0,1066
t_{13}	780	0,0639	0,1054	0,0708	0,1198	0,0681	0,1063
t_{14}	840	0,0641	0,1056	0,0707	0,1195	0,0680	0,1060
t_{15}	900	0,0641	0,1057	0,0707	0,1193	0,0678	0,1054
t_{16}	960	0,0643	0,1060	0,0704	0,1190	0,0678	0,1054
t_{17}	1020	0,0644	0,1062	0,0703	0,1188	0,0679	0,1052
t_{18}	1080	0,0648	0,1069	0,0703	0,1188	0,0678	0,1054
t_{19}	1140	0,0656	0,1077	0,0702	0,1185	0,0679	0,1044
t_{20}	1200	0,0662	0,1086	0,0702	0,1188	0,0680	0,1047
t_{21}	1260	0,0671	0,1095	0,0706	0,1191	0,0683	0,1029
$\Delta_{Abs}\% = \frac{(t0-t21)}{t0} \times 100$		Δ_{Abs}= 4%	Δ_{Abs}= 3%	Δ_{Abs}= 4%	Δ_{Abs}= 5%	Δ_{Abs}= 2%	Δ_{Abs}= 3%

Table S7 *UV-Vis absorbances at 234nm and 264nm of PLTX 1 μg/mL obtained at time zero (t_0) and after 21h (t_{21}) with relative variation (Δ Abs %) at 25±1°C.*

Time point	Time (min)	UV-Abs of PLTX 1 μg/mL at 25±1°C	
		50%MeOH	
		λ_{264nm}	λ_{234nm}
t_0	0	0,0105	0,0197
t_1	60	0,0105	0,0197
t_2	120	0,0109	0,0197
t_3	180	0,0110	0,0200
t_4	240	0,0110	0,0199
t_5	300	0,0110	0,0200
t_6	360	0,0109	0,0199
t_7	420	0,0111	0,0199
t_8	480	0,0110	0,0198
t_9	540	0,0112	0,0198
t_{10}	600	0,0109	0,0198
t_{11}	660	0,0110	0,0200
t_{12}	720	0,0111	0,0204
t_{13}	780	0,0111	0,0203
t_{14}	840	0,0112	0,0204
t_{15}	900	0,0110	0,0205
t_{16}	960	0,0111	0,0204
t_{17}	1020	0,0112	0,0203
t_{18}	1080	0,0112	0,0206
t_{19}	1140	0,0111	0,0206
t_{20}	1200	0,0111	0,0206
t_{21}	1260	0,0111	0,0206

$\Delta_{Abs}\% = \frac{(t0-t21)}{t0} \times 100$ Δ_{Abs}= -6% Δ_{Abs}= -5%

Table S8 *UV-Vis absorption values measured for PLTX 1 µg/mL at 6±1°C, λ_{max} 234 nm and 264 nm, time zero (t_0) and after 21h (t_{21}) with relative variation (Δ Abs %).*

**Time point**	**Time (min)**	**UV-Abs of PLTX 1 µg/mL at 6±1°C**	
		**50%MeOH**	
		λ_{264nm}	λ_{234nm}
t_0	0	0,0111	0,0164
t_1	60	0,0111	0,0164
t_2	120	0,0110	0,0164
t_3	180	0,0110	0,0164
t_4	240	0,0111	0,0164
t_5	300	0,0113	0,0163
t_6	360	0,0110	0,0161
t_7	420	0,0109	0,0163
t_8	480	0,0110	0,0162
t_9	540	0,0110	0,0162
t_{10}	600	0,0109	0,0162
t_{11}	660	0,0109	0,0163
t_{12}	720	0,0108	0,0162
t_{13}	780	0,0110	0,0162
t_{14}	840	0,0111	0,0161
t_{15}	900	0,0110	0,0162
t_{16}	960	0,0110	0,0161
t_{17}	1020	0,0110	0,0160
t_{18}	1080	0,0109	0,0160
t_{19}	1140	0,0110	0,0162
t_{20}	1200	0,0108	0,0161
t_{21}	1260	0,0108	0,0163

$\Delta_{Abs}\% = \frac{(t0-t21)}{t0} \times 100$ Δ_{Abs}= -3% Δ_{Abs}= -1%

Table S9 Procedure used for preparation of Sample #1-6.

Label	Procedure
**Sample #1 and #4**<br>PLTX 1 µg/mL in 10% MeOH<br>V= 200 µL	170 µL of pure water and 10 µL of MeOH were added to 20 µL of PLTX 10 µg/mL in 50% MeOH
**Sample #2 and #5**<br>PLTX 1 µg/mL in 50% MeOH<br>V= 200 µL	180 µL of 50% MeOH were added to 20 µL of PLTX 10 µg/mL in 50% MeOH
**Sample #3 and #6**<br>PLTX 1 µg/mL in 90% MeOH<br>V= 200 µL	170 µL of MeOH and 10 µL of pure water were added to 20µL of PLTX 10 µg/mL in 50% MeOH

Table S10 Procedure used for preparation of Sample #7-12.

Label	Procedure
**Sample #7 and #10**<br>PLTX 0.5 µg/mL in 10%MeOH<br>V= 200 µL	100 µL of 10% MeOH were added to 100 µL of PLTX 1 µg/mL in 10% MeOH
**Sample #8 and #11**<br>PLTX 0.5 µg/mL in 50%MeOH<br>V= 200 µL	100 µL of 50% MeOH were added to 100 µL of PLTX 1 µg/mL in 50% MeOH
**Sample #9 and #12**<br>PLTX 0.5 µg/mL in 90%MeOH<br>V= 200 µL	100 µL of 90% MeOH were added to 100 µL of PLTX 1 µg/mL in 90% MeOH

Table S11 Procedure used for preparation of Sample #13-15.

Label	Procedure
**Sample #13**<br>PLTX 10 µg/mL in 10% MeOH<br>V= 600 µL	480 µL of pure water were added to 120 µL of PLTX 50 µg/mL in 50% MeOH
**Sample #14**<br>PLTX 10 µg/mL in 50%MeOH<br>V= 600 µL	480 µL of 50% MeOH were added to 120 µL of PLTX 50 µg/mL in 50% MeOH
**Sample #15**<br>PLTX 10 µg/mL 90%MeOH<br>V= 600 µL	480 µL of MeOH were added to 120 µL of PLTX 50 µg/mL in 50% MeOH

Table S12 Procedure used for preparation of Sample #16-18.

Label	Procedure
**Sample #16**<br>PLTX 1 µg/mL in 10% MeOH<br>V= 600 µL	510 µL of water and 30 µL of MeOH were added to 60 µL of PLTX 10 µg/mL in 50% MeOH
**Sample #17**<br>PLTX 1 µg/mL in 50%MeOH<br>V= 600 µL	540 µL of 50% MeOH were added to 60 µL of PLTX 10 µg/mL in 50% MeOH
**Sample #18**<br>PLTX 1 µg/mL 90%MeOH<br>V= 600 µL	510 µL of MeOH and 30 µL of water were added to 60 µL of PLTX 10 µg/mL in 50% MeOH

Table S13 *Example of the procedure used to acquire UV-Vis spectra for Sample #13 and #14, namely PLTX 10* μg/mL *in 10% MeOH at 25°C and PLTX 10* μg/mL *in 50% MeOH at 25°C:*

(1) Baseline empty instrument
(2) Sample acquisition of two blank solvents:<br>Cell 1: **Blank 10%MeOH**<br>Cell 2: **Blank 50%MeOH**
(3) Baseline on cell 1 and cell 2
(4) Sample acquisition of solvents after baseline:<br>Cell 1: **Blank 10%MeOH_afterbaseline**<br>Cell 2: **Blank 50%MeOH_afterbaseline**
(5) Sample acquisition of PLTX samples #1 and #2:<br>Cell 1: **PLTX_10ppm_10%MeOH _25°C**<br>Cell 2: **PLTX_10ppm_50%MeOH _25°C**

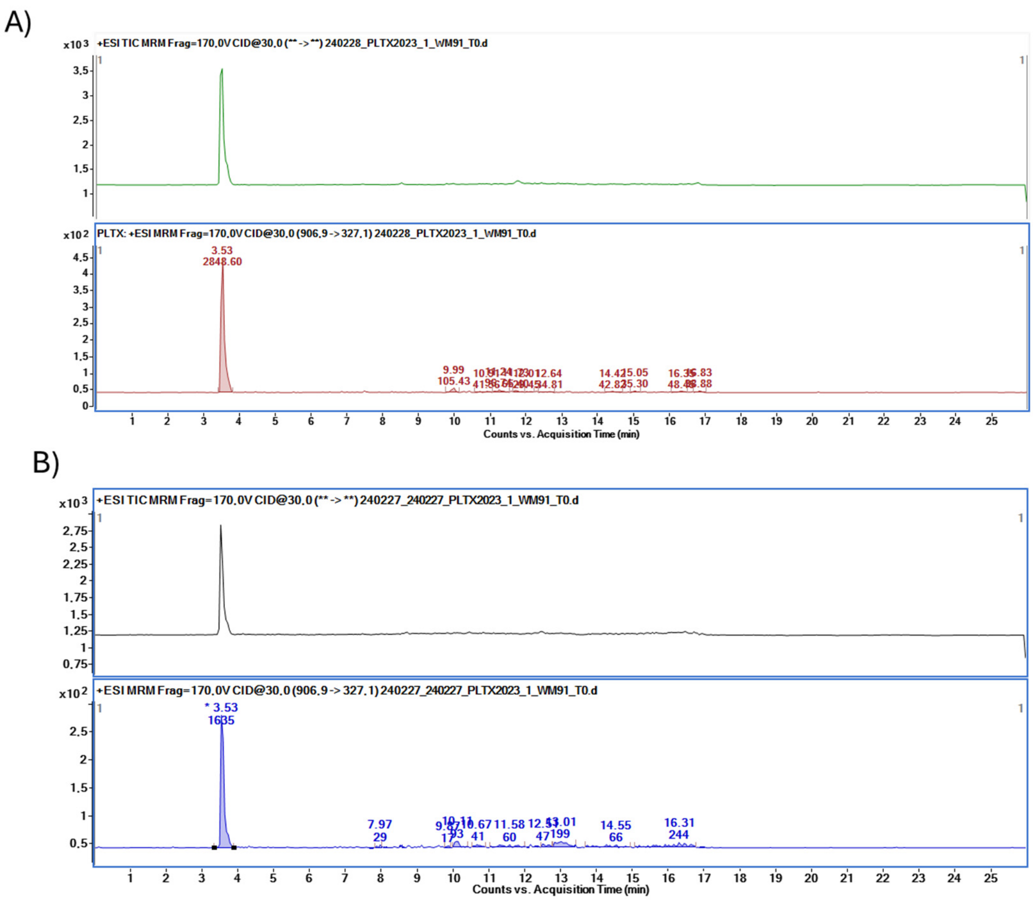

Figure S1 *Total Ion Chromatogram (TIC) and MRM quantifier transition (m/z 906.9à 327.1) for PLTX 1 µg/mL in 10%MeOH at* t_0 *at 25±1°C (A) and 6±1°C (B).*

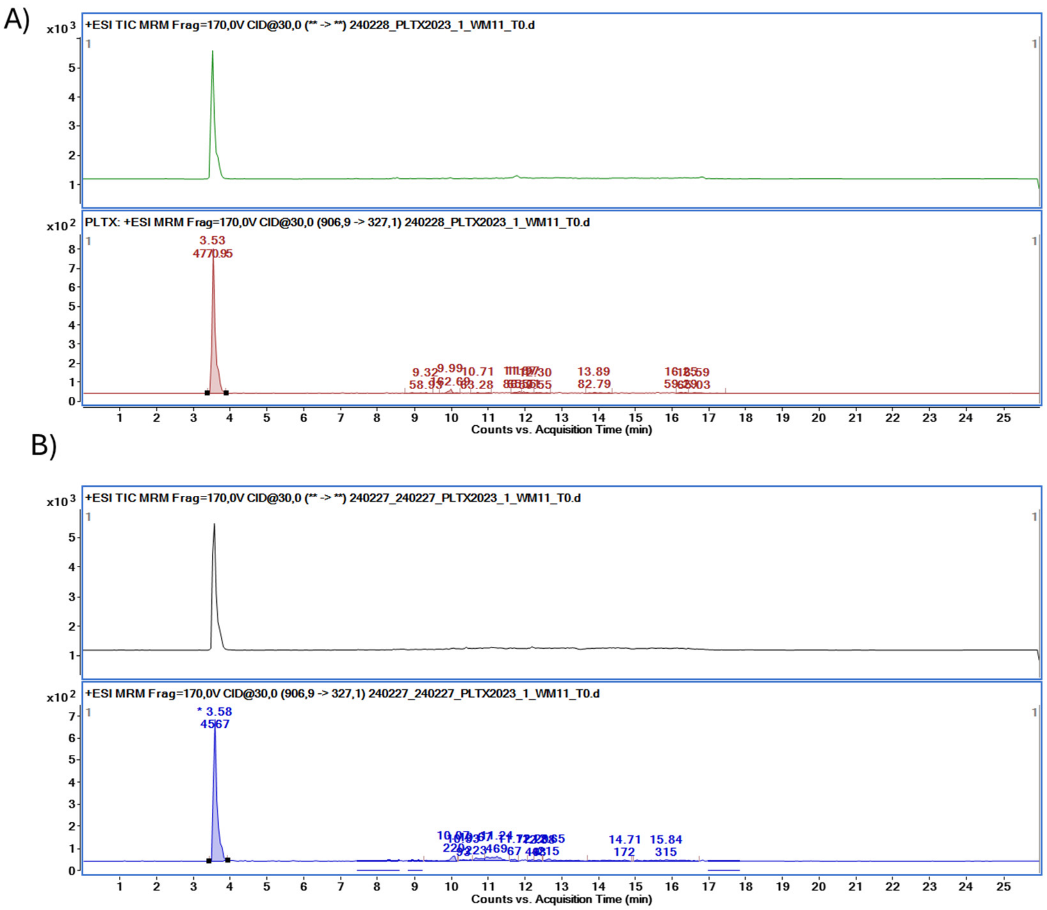

Figure S2 *Total Ion Chromatogram (TIC) and MRM quantifier transition (m/z 906.9à 327.1) for PLTX 1 µg/mL in 50%MeOH at* t_0 *25±1°C (A) and 6±1°C (B).*

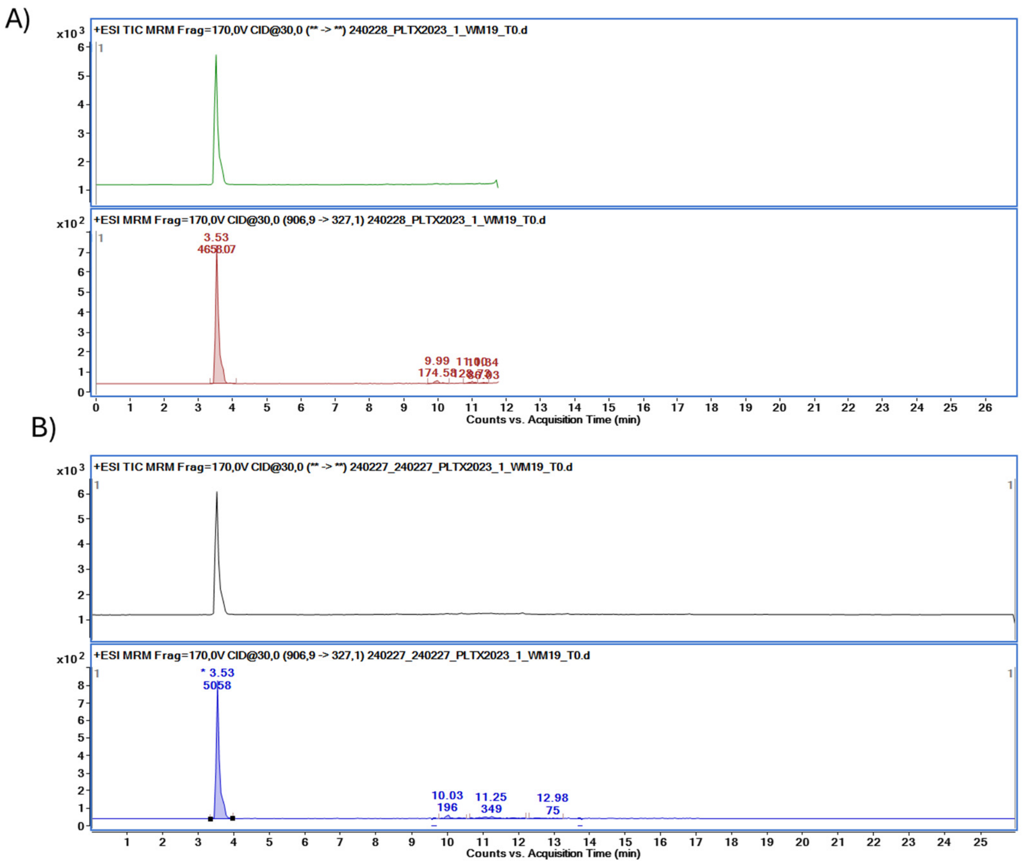

Figure S3 *Total Ion Chromatogram (TIC) and MRM quantifier transition (m/z 906.9à 327.1) for PLTX 1 µg/mL in 90%MeOH at* t_0 *25±1°C (A) and 6±1°C (B).*

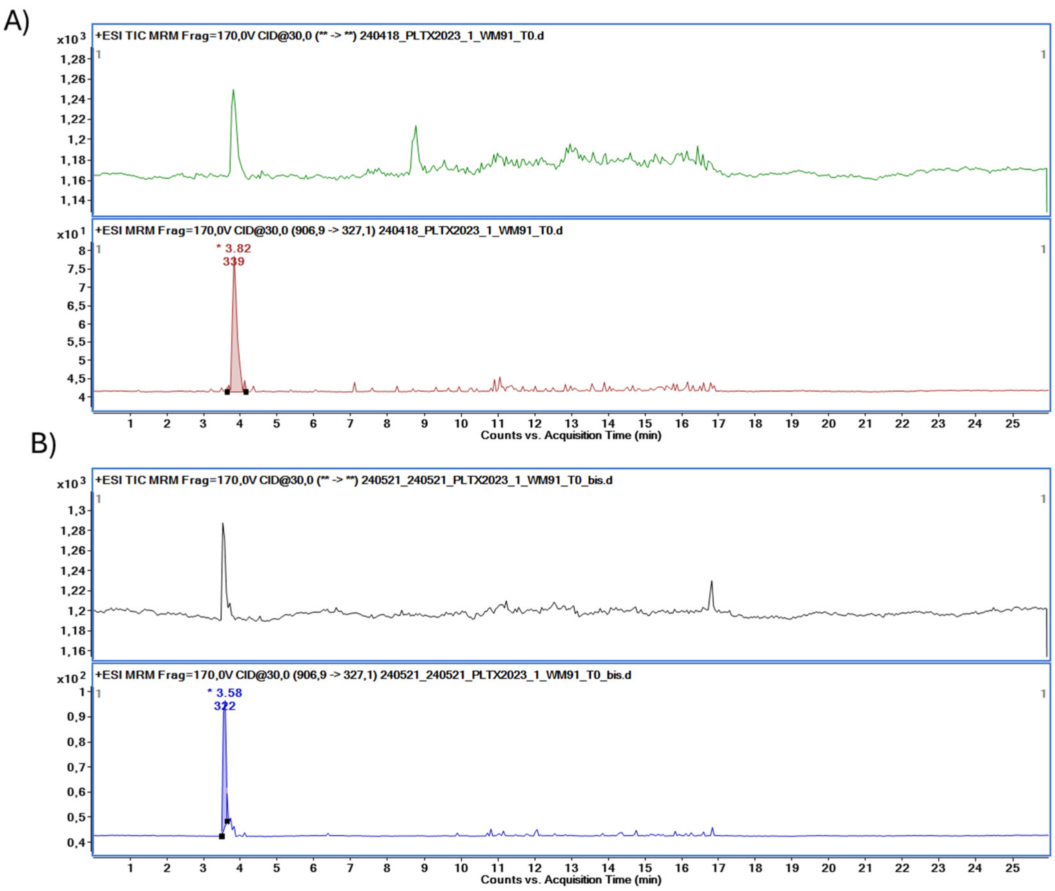

Figure S4 *Total Ion Chromatogram (TIC) and MRM quantifier transition (m/z 906.9à 327.1) for PLTX 0.5 µg/mL in 10%MeOH at* t_0 *25±1°C (A) and 6±1°C (B).*

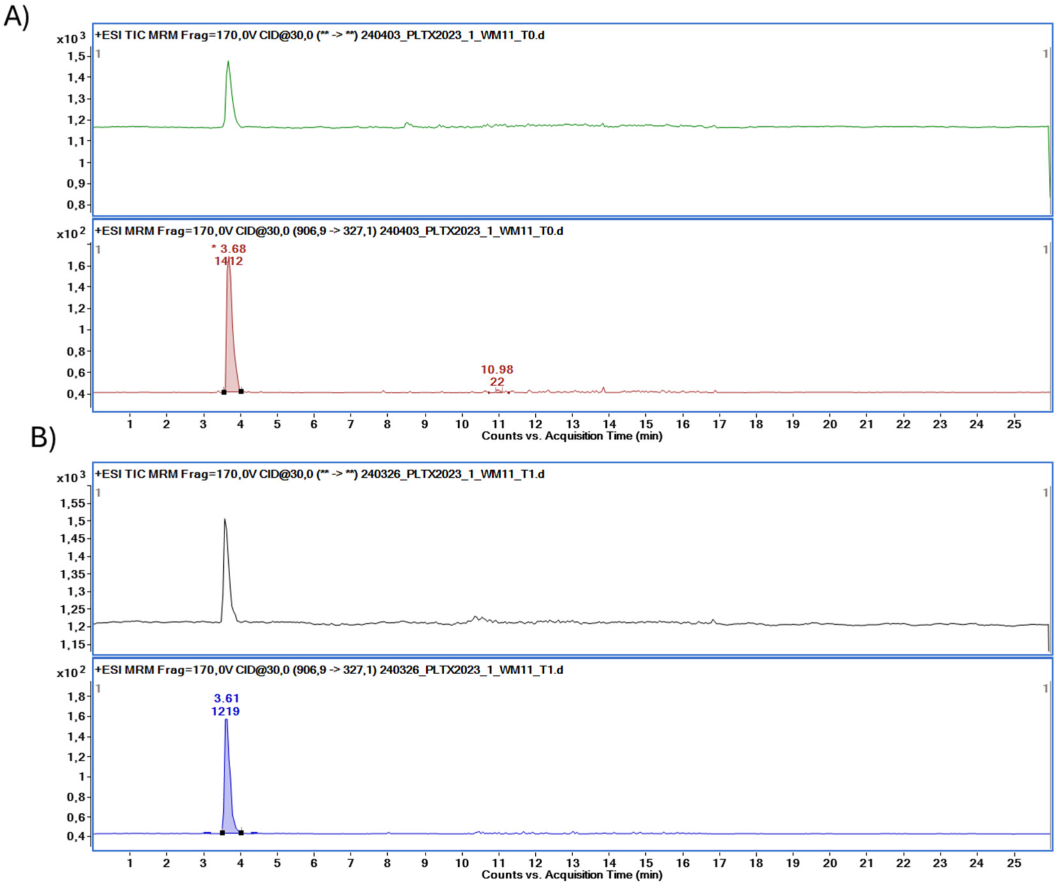

Figure S5 Total Ion Chromatogram (TIC) and MRM quantifier transition (m/z 906.9à 327.1) for PLTX 0.5 µg/mL in 50%MeOH at t_0 25±1°C (A) and 6±1°C (B).

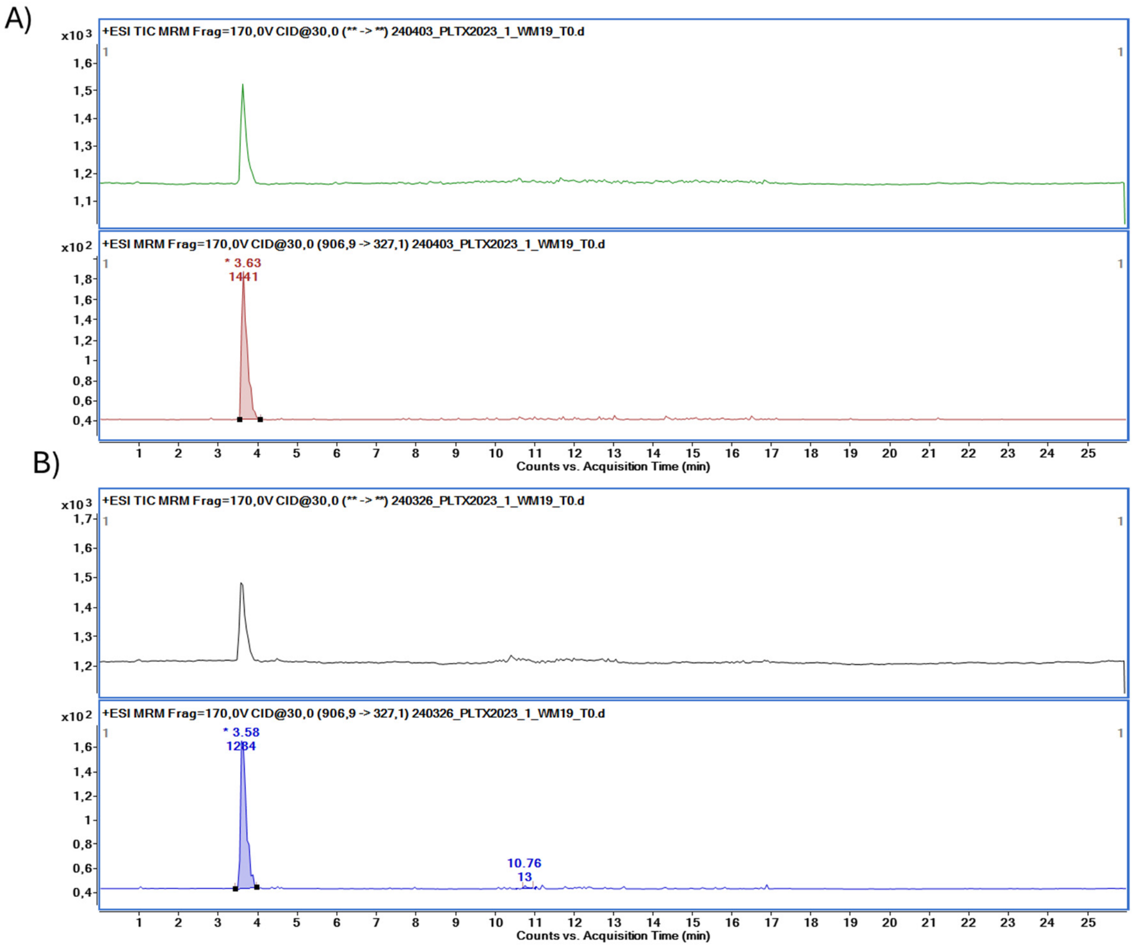

Figure S6 Total Ion Chromatogram (TIC) and MRM quantifier transition (m/z 906.9à 327.1) for PLTX 0.5 μg/mL in 90%MeOH at t_0 25±1°C (A) and 6±1°C (B).

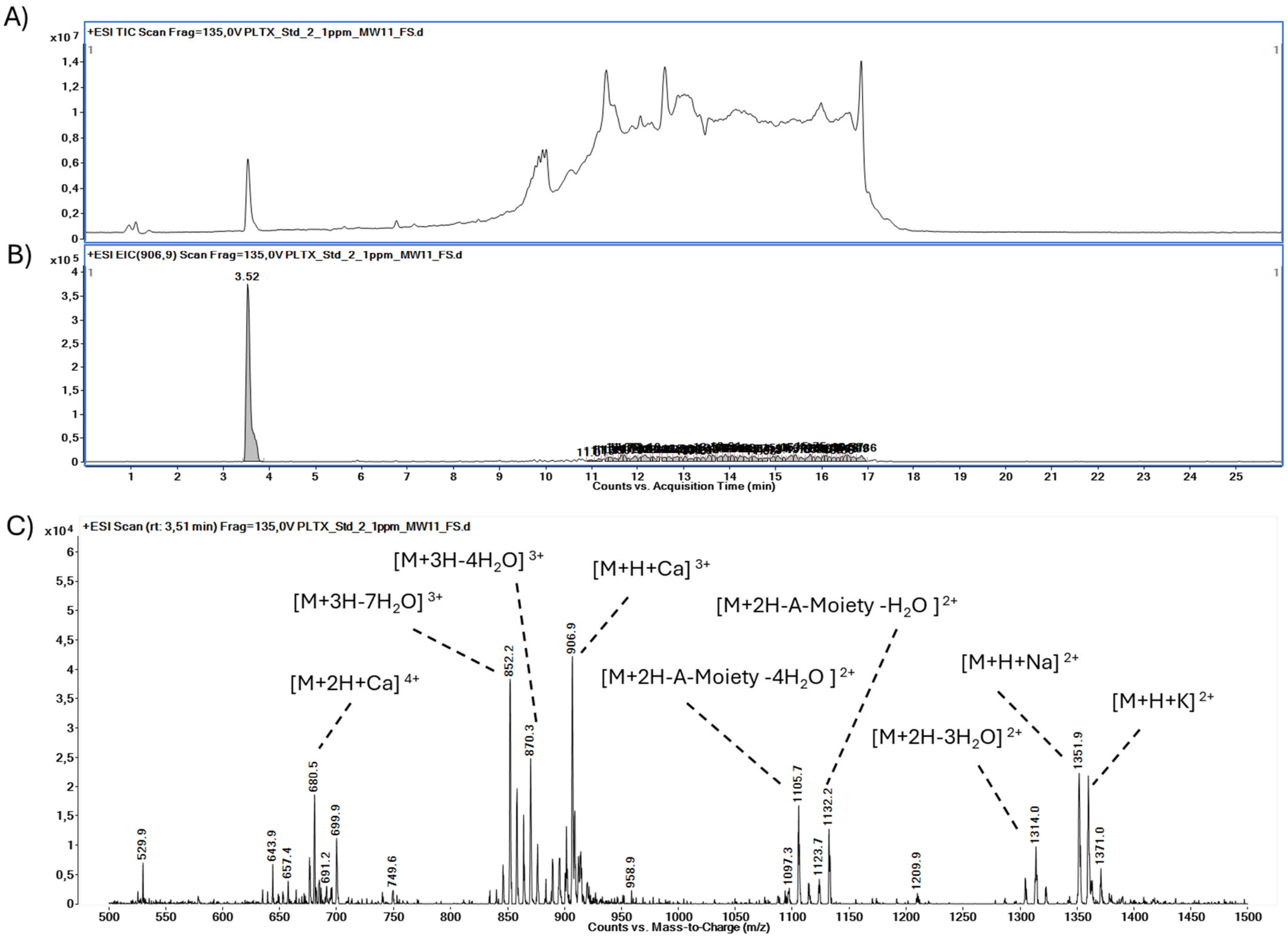

Figure S7 *LC-MS of PLTX 1 µg/mL in 50% MeOH acquired in full scan MS positive ion mode in the mass range m/z 500-1500, scan time 500 ms, fragmentor 135 V, accelerator voltage 5 V. (A)Total ion chromatogram (TIC), (B) extracted ion chromatogram (EIC) of [M+H+Ca]* $^{3+}$ *ion of palytoxin at m/z 906.9 and (C) full Scan MS spectrum associated to the peak eluting at 3.5 min with ion assignment of the main ions.*
